# Biosynthesised Silver Nanoparticles Loading onto Biphasic Calcium Phosphate for Antibacterial and Bone Tissue Engineering Applications

**DOI:** 10.3390/antibiotics11121780

**Published:** 2022-12-08

**Authors:** Varun Prasath Padmanabhan, Pugalmani Sivashanmugam, Ravichandran Kulandaivelu, Suresh Sagadevan, Balu Sridevi, Rajakumar Govindasamy, Muthu Thiruvengadam

**Affiliations:** 1 Department of Analytical Chemistry, University of Madras, Guindy Campus, Tamil Nadu, Chennai 600025, India; 2 Department of Orthodontics, Saveetha Dental College and Hospitals, Saveetha Institute of Medical and Technical Sciences, Saveetha University, Tamil Nadu, Chennai 600077, India; 3 Nanotechnology & Catalysis Research Centre, University of Malaya, Kuala Lumpur 50603, Malaysia; 4 Department of Electronics and Communication Engineering, Velammal Institute of Technology, Ponneri, Tamil Nadu, Thiruvallur 601204, India; 5 Department of Applied Bioscience, College of Life and Environmental Sciences, Konkuk University, Seoul 05029, Republic of Korea

**Keywords:** biphasic calcium phosphate, Ag nanoparticles, antibacterial activity, hydroxyapatite, β-tricalcium phosphate, MTT assay

## Abstract

Biphasic calcium phosphate (BCP) serves as one of the substitutes for bone as it consists of an intimate mixture of beta-tricalcium phosphate (β-TCP) and hydroxyapatite (HAP) in different ratios. BCP, because of its inbuilt properties such as osteoconductivity, biocompatibility, and biostability in several clinical models serves as a bone substituent for orthopedic applications. Therefore, the present study aimed to assess the effectiveness of silver (Ag) nanoparticles (NPs) combined with BCP composites for the orthopedic sector of bone tissue regeneration and growth. In this regard, we first synthesized Ag-BCP microclusters by the double-emulsion method and then characterized the composite for various physicochemical properties, including the crystallinity and crystal structure, bonding and functionality, porosity, morphology, surface charges, topography, and thermal stability. In addition, the antibacterial activity of Ag-BCP was tested against gram-positive and gram-negative microorganisms such as *Staphylococcus aureus*, *Candida albicans*, and *Escherichia coli*. Finally, the cytocompatibility of Ag-BCP was confirmed against the fibroblast cells in vitro.

## 1. Introduction

In recent years, research relating to stem cells and tissue engineering has produced efficacious therapeutic strategies for the treatment of damaged bones and their cells through the regeneration/remodeling pathways [1]. For such applications, the composite scaffolds containing the ingredients such as polymers [2,3], ceramics [4], metal nanoparticles (NPs), and their composites [5] are highly suitable because of their inbuilt properties such as porosity, conductivity, resistance, and biocompatibility. These synthetic composites with their porous and biocompatible nature provide a suitable environment for regeneration with complete functionality and effective proliferation of cells that eventually replace the diseased bone cells. Furthermore, the synthetic bone scaffolds made up of said ingredients have the capacity to encapsulate the therapeutic drug molecules that are useful for the treatment of commonly attacking orthopedic diseases like bone and bone marrow tumors, osteoporosis, and to avert an infection [6]. Of the various ingredients, the bioceramic material, biphasic calcium phosphate (BCP) has several interesting properties and the most important is its mineral portion which is relatively easy to process and has an excellent cell–cell attachment capacity. All these properties support its incorporation as a bone substituent, biocement, surface coating, drug-delivery platform, and tissue engineering scaffold [7,8,9]. Additional advantages of BCP material are the low cost, unlimited availability, biocompatibility, predictability, biosafety, and lower morbidity to the patient over autografts and allografts. Hence, this material serves as an attractive option for bone tissue engineering, dental replacements, craniofacial surgeries, spinal surgery, and neurosurgeries [10].

The properties of BCP materials (similar to many different composites) can be strongly influenced by altering the production parameters such as the solution pH, sintering temperature, and purification processes. The calcium phosphates (Ca_3_PO_4_) thus formed consist of unique physicochemical characteristics like an altered surface area, porosity, surface energy, charges, and roughness [11,12,13]. In this direction, to control the pore sizes of bioceramic compounds, one approach involves the incorporation of porogens and pore-formers. An alternative method to this approach is the application of heat treatment to generate macropores (diameter >100 μm) and micropores (with <10 μm diameter) [14]. Therefore, taking advantage of this heat-induced method for the formation of high-surface bioceramics having a macroporous and microporous nature to suit osteoconductivity, many researchers have demonstrated the role of BCP as a bone substituent [15,16]. Moreover, the adjacent concavities and nearby walls of the macropores serve as a salient point and favor the formation of geometric-dependent of bone [17,18]. Furthermore, the surface dissolution leads to the supersaturation of calcium (Ca) and phosphate (PO_4_^3−^) ions, causing reprecipitation and the generation of a biocompatible surface layer that permits an easy bonding of bone with the synthetic bioceramic. This process has an impact on the potential of osteoinduction [19,20]. Of various kinds of Ca_3_PO_4_, the BCP kind is made up of stable and soluble phases of ions with varying concentrations. Furthermore, the other form, hydroxyapatite (HAP; chemical formula Ca_10_(PO_4_)_6_(OH)_2_), would be advantageous on top of other calcium phosphates because of the guided bioactivity in linking the resorption/solubilization and biomaterial stability towards the promotion of bone growth [21,22].

Silver (Ag) nanoparticles (NPs) are widely used in the treatment of bacterial infections associated with injuries, wounds, tissue engineering and in the water treatment sector [23]. Ag NPs have high surface charges, surface area, surface-to-volume ratio, and surface oxygen defects that promote antibacterial activity in contrast to the other Ag-salts and organometallics [24,25]. Nevertheless, the stability and dispersion of Ag NPs curb their biological efficiency by aggregation that leads to the formation of larger-sized crystals and decreases the cumulative surface area. In some instances, this aggregation is overcome to stabilize the NPs on substrates, leading to stability enhancement and an associated antibacterial effect.

By considering the potential advantages of BCP to serve as a bone substituent and Ag NPs for the impending antibacterial activity, the present work aimed to develop a nanocomposite that has multiple functions to suit bone tissue engineering applications. For that, we fabricated a hybrid composite consisting of a BCP matrix decorated in situ with that of Ag NPs and for the formation of the composite, we employed a facile double-emulsion method. The nanocomposite was analyzed for its physicochemical properties by making use of various instrumental techniques like FTIR, powder XRD, SEM, zeta potential, surface topology and TGA-DTG. Further, we tested the controlled drug release, antibacterial activity, and in vitro cell viability capacity of as-synthesized Ag-BCP nanocomposite.

## 2. Materials and Methods

### 2.1. Formation of Ag NPs

About 50 mL of 0.01 mM AgNO_3_ in an aqueous solution was mixed with 50 mL of 0.1 mM glucose solution and the reaction was maintained at a pH of 11 using ammonia. The reaction was kept for aging for 6 h and until the solution color changed from colorless to yellow, confirming the formation of Ag NPs. The Ag NPs were separated by centrifugation, washed with ethanol 2–3 times, dried, and stored for loading onto BCP.

(1) $$C_{6}H_{12}O_{6}+2{\mathrm{AgNO}}_{3}\;\overset{2NH_{4}OH}{\to }\;\mathrm{Gluconic}\;\mathrm{acid}+2\mathrm{Ag}+2{\mathrm{NH}}_{4}{\mathrm{NO}}_{3}+H_{2}O$$

### 2.2. Synthesis of Ag-Decorated BCP

For the fabrication of the Ag-BCP nanocomposite, we first formed BCP by the combination of hydroxyapatite (HAP) and β-tricalcium phosphate (β-TCP). Both were prepared individually. HAP was first prepared by mixing 50 mL of 1 M Calcium Nitrate as Ca precursor solution (adjusted to a pH of 11 using NH_4_OH) with 50 mL of 0.66 M phosphate solution in a dropwise manner. After the complete addition, the milky-white-colored solution was stirred constantly for another 2 h to generate a white precipitate which was kept for 24 h. Then, the precipitate was separated by filtration and washed with a solvent mixture containing a 1:2 ratio of ethanol to water. The precipitate was kept in an oven furnace for sintering at 800 °C overnight to finally generate a white powder of HAP.

For the formation of β-TCP, a simple co-precipitation method was used. To this end, 50 mL of an aqueous solution of calcium nitrate (0.9 M) was added dropwise to 50 mL of ammonium dihydrogen phosphate (0.6 M) at a pH of 8 maintained by using concentrated ammonia. Here, the Ca/P ratio of 1.5 was retained manually and the magnetic stirring (set in the range of 200–250 rpm) was continued for 2 h even after the complete addition. After that, the precipitate was separated, washed with an ethanol-water mixture, dried in an oven, crushed, and sintered at 900 °C in a muffle furnace for 1 h. The final product was stored in an airtight container.

For the synthesis of BCP implants [26], HAP and β-TCP powders in a 60:40 ratio were grounded homogeneously using a mortar and pestle. The finely powdered mixture was initially dried at 55 °C overnight and further subjected to 100 °C for 5 h. The fully dried powder was collected and sealed with a polythene cover until its use. Finally, for the loading of Ag NPs onto BCP, individual aqueous solutions containing equal amounts of Ag NPs (25 mL) and BCP (25 mL slurry) were added together. The mixture was subjected to ultrasonication to undergo homogenous mixing for a period of 15 min. This resulted in the formation of a light-yellow-colored viscous solution that was kept in an oven at 110 °C for drying. The dried powder of the Ag-BCP composite was collected and used for further analysis.

### 2.3. Instrumentation

Powdered X-ray diffraction (XRD) analysis was carried out to understand the crystal structure and crystallinity. The powder samples were run in the 2θ range of 20–80° (Model: Smart Lab se X-ray, Rigaku, Japan; k = 1.5418 Å). The morphology of the samples was investigated using a field emission scanning electron microscopy (FESEM) connected with an energy-dispersive X-ray diffractometer (Model: JOEL JFM 6390 Scientific, Peabody, MA, USA). The functionality and bonding of samples were studied using Fourier transform infrared spectroscopy (FTIR) in the wavenumber range of 4000–400 cm^−1^ (Spectrum 2, PerkinElmer, Waltham, MA, USA) and confocal Raman spectroscopy (Alpha 300r, Witech, Braunschweig, Germany). Furthermore, atomic force microscopy (AFM) studies were employed to investigate the surface nature of the samples (Park xe7, Park system, Suwon, Republic of Korea). The elemental composition of the samples was analyzed using X-ray photoelectron spectroscopy (XPS, ULVAC-PHI, Inc; Model: PHI5000 Version Probe III). For the thermal stability and phase changes, thermogravimetric analysis (TGA) and differential thermal analysis (DTA) were performed (Netzsch sta 2500 was measured under N_2_ environment between 30–800 °C). The zeta potential and dynamic light scattering analysis were used to determine the particle size and surface charges (Horiba Scientific Sz-100 instrument).

### 2.4. Measurement of Porosity

To investigate the porosity of pelletized BCP and Ag-BCP samples, the liquid displacement technique was used. The pelletized samples of BCP and Ag-BCP are not soluble in ethanol. Thus, the penetrating ability of ethanol into the sample pores restricts the occurrence of any swelling or shrinkage. For the testing, the sample pellet with a known weight (W) was first immersed in a graduated cylinder that already had a known volume (V_1_) of ethanol. The emigration (of ethanol) followed by depressurization for the undisturbed samples can be seen. This diffusion of ethanol into the pores can be continued until we see the halting of air bubbles and at this stage, the volume of ethanol (i.e., sample pellet soaked in ethanol) was noted as V_2_ and we measured the difference in two volumes (V_2_–V_1_). Further, the sample pellet was removed from the cylinder containing ethanol and we measured the residual volume (V_3_) of ethanol. By making use of W, V_1_, V_2_, and V_3_ in the following formula, the porosity was calculated.

(2) $$\mathrm{Porosity},\;\varepsilon =\frac{\left(V1-\;V3\right)}{\left(V2-\;V3\right)}$$

### 2.5. In-Vitro Bioactivity and Biodegradation Studies

To investigate the extent of bioactivity for our materials in stimulated fluid (SBF) in vitro, we first prepared a solution consisting of NaCl (7.9 g), NaHCO_3_ (0.3 g), KCl (0.2 g), K_2_HPO_4_.3H_2_O (0.2 g), MgCl_2_.6H_2_O (0.3 g), CaCl_2_ (0.2 g), Na_2_SO_4_ (0.07 g) and (CH_2_OH)_3_CNH_2_ (6.0 g) in double-distilled water (added one after the other). The formed solution mixture pH was set to 7.4 with the help of HCl and maintained at 37 °C. For the testing, the pelletized samples of BCP and Ag-BCP were soaked in SBF for 21 days and after that, removed, rinsed with de-ionized water, and further subjected to SEM analysis to investigate the extent of biomineralization (formation of any mass) at the surface.

The in vitro biodegradation/biodissolution studies were performed by investigating the amount of Ag and Ca ions that were released into the buffer solution and by recording the morphological changes linked to the release. The pellet (with a weight of 0.5 g as W_0_) made from the granules was first immersed in a 20 mL tris-buffer solution maintained at a pH of 7.4 at 37 °C. At the end of 3 weeks of incubation, we rinsed the sample pellets with ethanol, dried, and measured its final mass (W_t_). The weight loss can be calculated using the equation:

Weight decrease = (W_t_/W_0_) × 100% (3)

### 2.6. Studies of Drug Loading and Release

The drug loading capacity and release efficiency from the BCP and Ag-BCP matrices were evaluated using the typical drug, Ciprofloxacin (CIP). Typically, 10 mg of CIP was dispersed in 100 mL of double-distilled water containing 90 mg either of BCP or Ag-BCP. The mixture was allowed to stir for 24 h at room temperature. Then, the precipitate was separated by centrifugation, rinsed with distilled water, and dried. The drug-loaded sample in the form of a pellet was collected. Further, for the drug release studies, a known weight of bioceramic pellet loaded with CIP was placed in phosphate-buffered saline (PBS; pH 7.4) and subjected to horizontal agitation on a shaking water bath set at 37 °C. After each specified interval of time, about 5 mL of the sample (containing the released CIP) was collected and replaced with an equal amount of fresh medium. UV–Vis spectrometry was used for the qualitative and quantitative investigation of released CIP at various time intervals.

### 2.7. Antibacterial and Antifungal Activity

The antibacterial activity of our bioceramic samples was measured by the agar disc diffusion method involving Muller Hinton Agar (MHA) medium. The stock cultures were maintained at 4 °C on the slant of the nutrient agar. The active cultures were prepared by transferring a loop full of bacterial cells from the cultured stocks to nutrient broth-loaded test tubes and subjected to incubation at 37 °C for 24 h. The strains were used for their antibacterial activity against the Gram-positive *S. aureus*, Gram-negative *E. coli*, and antifungal activity against *C. albicans*. The discs were prepared with 20 µL of each of the samples (Ag-BCP, HAP, β-TCP, and BCP), a negative control of dimethylsulfoxide (DMSO), and a Standard 1 mg/mL of Streptomycin as positive control). The plates were incubated for another 24 h at 37 °C and, finally, the growth of microbes was investigated by measuring the diameter of the zone of inhibition (ZoI).

For the antifungal activity, the assay procedure is almost the same. We used the agar disc diffusion method, where the stock cultures were maintained at 4 °C on the slant of potato dextrose agar (PDA). Briefly, 4.4 g of PDA was weighed and dissolved in 100 mL of distilled water followed by the addition of 1 g of agar. Then, we subjected the media to sterilization, solidified the media for 1 h, and spread the inoculums on solid plates with a sterile swab moistened with the fungal suspension. The discs contained 20 µL of each of the testing samples (Ag-BCP, HAP, β-TCP, and BCP), a negative control (DMSO), and a positive control (1 mg/mL of Ketoconazole). The extent of antifungal activity was measured by incubating the sample-treated plates at 37 °C for 24 h and finally recording the diameter of ZoI in mm.

### 2.8. In Vitro Cell Viability Assay

To investigate the cytocompatibility performance of as-synthesized bioceramic composites, in vitro cell viability studies were carried out on the L929 mouse fibroblast cell line over a 24 h period. Briefly, 1 × 10^5^ cells per well were added to a 96-well plate containing Dulbeccos Modified Eagle Medium (DMEM) and 10% fetal bovine serum (FBS). The cells were allowed to grow until reaching their confluency level. Then, they were washed with a fresh serum-free medium 2–3 times, followed by starvation for 1 h at 37° C. Subsequently, the cells were treated with different concentrations (31.2–1000 mg/mL) of bioceramics, BCP, and Ag-BCP over a 24-h period. Then, the old medium was replaced with a fresh serum-free medium comprising 0.5 mg/mL of MTT (3-[4,5-dimethylthiazol-2-yl] 2,5-diphenyl tetrazolium bromide) and incubated for another 4 h at 37 °C in a CO_2_ incubator. The medium containing MTT was removed and the cells were washed with PBS to eliminate any unreacted reagent. Then, we added DMSO while thoroughly mixing by pipetting up and down to dissolve the formed formazan crystals. Finally, the purple-blue-colored formazan crystals were analyzed spectrophotometrically by recording the absorbance at 570 nm (Biorad 680). Using these readings, the cytotoxicity was determined using the Graph pad prism 5 software. Furthermore, the viable L929 cells were observed using inverted phase-contrast microscopy.

Percentage (%) of cell viability = (Sample’s OD/Control’s OD) × 100 (4)

### 2.9. Statistical Analysis

All the statistical analyses were performed using a one-way analysis of variance (ANOVA) and the data presented are the mean ± standard deviation of at least three individual experiments with the value of *p* < 0.05 as statistically significant.

## 3. Results and Discussion

### 3.1. Physicochemical Analysis

**Figure 1:**
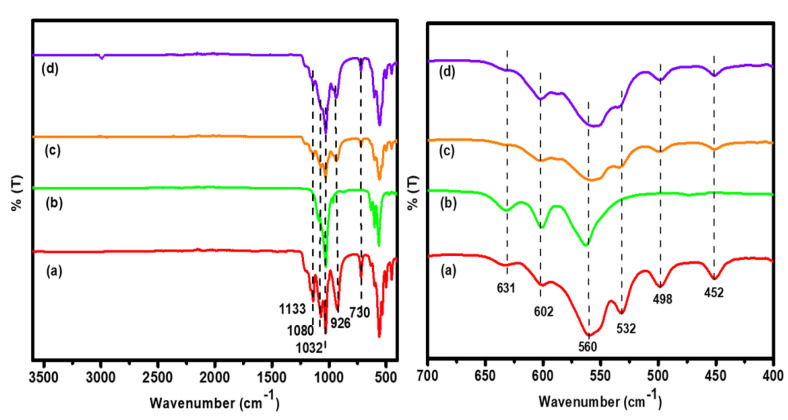
Comparison of the FTIR spectra of (**a**) HAP, (**b**) β-TCP, (**c**) BCP, and (**d**) Ag-BCP samples.

Figure 1 shows the FTIR spectral analysis of (a) HAP, (b) β-TCP, (c) BCP, and (d) Ag-BCP samples. All spectra indicate the presence of characteristic bands at 1032, 1098, and 1133 cm^−1^ due to the triply degenerated (υ_3_) asymmetric stretching vibrations of P-O bonds. Furthermore, the observation of bands at 602 and 560 cm^−1^ signifies the υ_4_ vibration of -PO_4_ group and the band at 926 cm^−1^ indicates the υ_1_ vibration of phosphate bond. The bending vibrational band observed at 630 cm^−1^ infers the liberational -OH group (due to surface adsorbed water vapor/moisture) and the bands at 498 and 452 cm^−1^ are assigned to the υ_2_ vibration of the PO_4_^3−^ group. The asymmetric bending vibrations of the phosphate group present in HAP were evident through the observation of a band at 608 cm^−1^. Similarly, for the β-TCP sample, some prominent sharp bands were observed at 560 and 602 cm^−1^ and can be linked to the bending vibrations of PO_4_^3−^ group. The band at 960 cm^−1^ (visible as a minor hump) stems from the υ_1_ frequency of vibration, and the band at 1037 cm^−1^ from the P-O stretching vibration of PO_4_^3−^ ions in β-TCP [27]. Further, for each sample, we observed some prominent bands at 3572 and 630 cm^−1^ owing to the presence of hydroxyl groups, i.e., the sharp band at 730 cm^−1^ is due to H_2_O (surface adsorbed) is available in all the tested samples, indicating the presence of moisture. For the BCP sample, the υ_1_ and υ_4_ absorption bands were observed at 926 and 567 cm^−1^, respectively. The intensity of the band observed at 926 cm^−1^ was least pronounced for BCP and Ag-BCP samples and was due to the composition of the 60:40 ratio of HAP and β-TCP. We observed no evidence for the occurrence of any chemical bonding between BCP and Ag, meaning that the Ag NPs were physically embedded in the BCP. From the analysis, the band patterns that appeared in all the samples (a-d) are best correlated with the earlier reports. Table 1 shows the composition of FT-IR spectra of all the samples.

**Figure 2:**
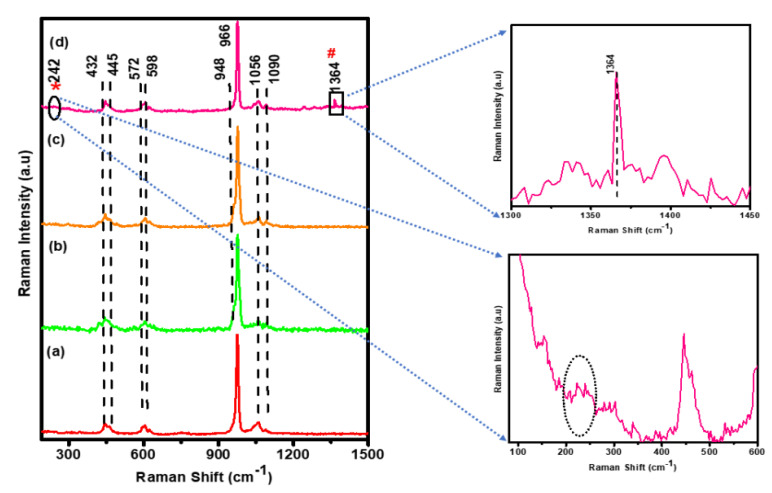
Comparison of the Raman spectra of (a) HAP, (b) β-TCP, (c) BCP, and (d) Ag-BCP samples.

Figure 2 compares the Raman spectroscopic analysis of (a) HAP, (b) β-TCP, (c) BCP, and (d) Ag-BCP samples. Table 2 shows the Raman spectral composition of all the samples. The band with the highest intensity around 966 cm^−1^ relates to the symmetrical stretching vibrations (υ_1_) of the phosphate (PO_4_^−3^) confirming the formation of HAP. Furthermore, for the same HAP sample, the symmetrical (υ_2_) and antisymmetrical bending (υ_4_) vibrations of the PO_4_^−3^ groups appeared at 432, 445, 572, and 598 cm^−1^, respectively. Additionally, the asymmetric stretching vibrations (υ_3_) of PO_4_^−3^ ions in the HAP were observed as weak intensity bands around 1056 and 1090 cm^−1^ [28]. The high-intensity band at 964 cm^−1^ besides a weak shoulder at 948 cm^−1^ indicates the internal vibrations of β-TCP in the BCP sample. For the BCP sample shown in Figure 2c, the spectrum has the same major band at 966 cm^−1^ and a shoulder band at 948 cm^−1^ corresponding to the β-TCP phase, as the BCP is composed of HAP and β-TCP in a 60:40 ratio. Finally, for the Ag-BCP sample (Figure 2d), the Ag NPs were loaded onto the BCP composite. The Ag particles were synthesized using glucose and thus in this reaction gluconic acid is formed as a by-product which is observed as a minor band at 1364 cm^−1^ [29]. Furthermore, the minor band at 226 cm^−1^ corresponds to the presence of Ag NPs.

**Figure 3:**
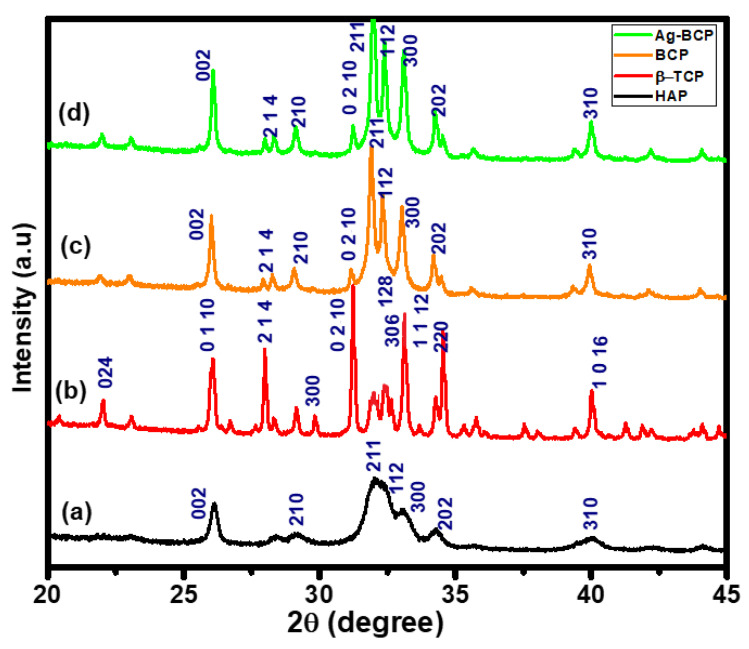
Powder XRD analysis for (a) HAP, (b) β-TCP, (c) BCP, and (d) Ag-BCP.

The powder XRD reflection patterns of the four bioceramics (HAP, β-TCP, BCP, and Ag-BCP) provided in Figure 3 confirm the formation of highly crystalline phases in all samples, as shown by the narrow and sharp pattern. The XRD patterns provided in Figure 3a,b show the calcium phosphate precursors derived from the HAP sintered at 800 °C and β-TCP at 900 °C, respectively. From the comparative analysis, the HAP patterns have perfectly matched with the parent HAP, as provided by the JCPDS card No. 09–0432. The crystalline nature of the HAP sample was confirmed by the patterns observed at 2θ of 25.9° (002), 28.6° (210), 31.7° (211), 32.2° (112), 34.0° (202), and 39.8° (310). These diffraction patterns indicate the presence of HAP in BCP (JCPDS No. 9-432). The calcium to phosphorous (Ca/P) ratio was found to be 1.6. Similarly, the reflection patterns of the β-TCP sample (Figure 3b) appeared at 2θ of 21.8° (024), 25.8° (1010), 27.8° (214), 31.0° (0210), 32.4° (128), and 34.3° (220) (JCPDS No. 9-169), with the Ca/P ratio being 1:5. Hence, all the diffraction patterns of HA and β-TCP in BCP were found to be more distinct and thereby indicate the crystalline nature. Additionally, the XRD patterns of BCP (Figure 3c) and Ag-BCP (Figure 3d) were observed at 2θ of 31.76° (211), 32.15° (112), 32.89° (300), and 34.02° (202), with the Ca/P ratio of 1:6. These data also confirm the formation of BCP in its highly crystalline phase. It is evident from the FTIR spectrum (Figure 1a–c) and the XRD phase composition (Figure 3c,d) that there is no formation of a Calcite (calcium carbonate) pattern at 29.4°. Moreover, it can be observed from the XRD pattern that the characteristic patterns of HAP in the BCP sample are much greater than in β-TCP [30]. Finally, the XRD pattern of the Ag-BCP sample (Figure 3d) is in good agreement with that of BCP (Figure 3c). Furthermore, the observation of no additional patterns for Ag NPs indicates its lower concentration.

**Figure 4:**
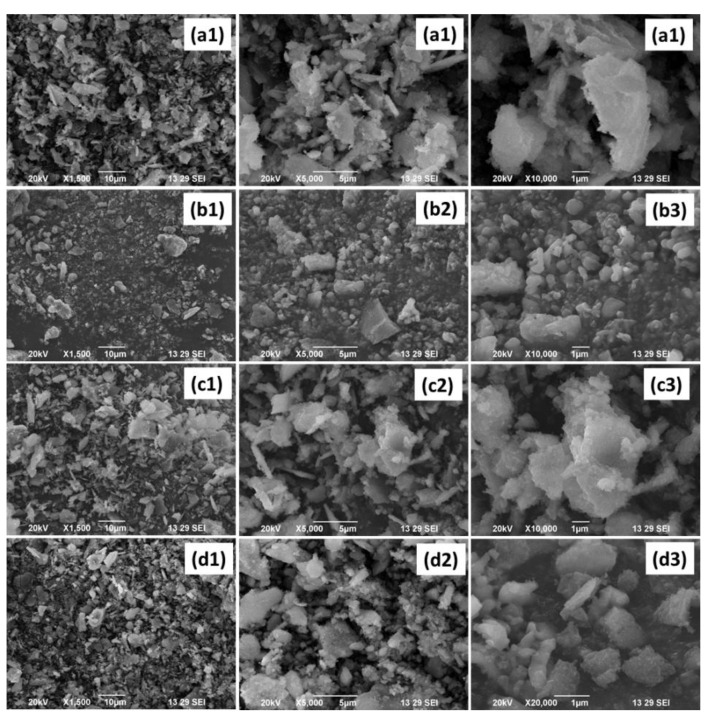
FESEM analysis of (**a**) HAP, (**b**) β-TCP, (**c**) BCP, and (**d**) Ag-BCP.

Figure 4 represents the FESEM analysis of (a) HAP, (b) β-TCP, (c) BCP, and (d) Ag-BCP at three different magnifications. The surfaces of all samples appear to be rough and granular and the maximum effect can be seen in β-TCP. The FESEM of HAP (Figure 4a1) showed that there is a decreased surface roughness. Furthermore, the FESEM of β-TCP (Figure 4b1) shows the micro- and macropores with a well-organized pore network, which supports its enhanced solubility effects. Furthermore, this kind of pore arrangement is predicted to permit the uptake of fluid, cell accommodation, and a greater surface area. The FESEM of BCP (Figure 4c1) showed less roughness and the surface is uneven with its patterns similar to the earlier report [27]. Finally, the morphology of Ag-BCP provided in Figure 4d1 shows some small decorating particles and are referred to as Ag NPs.

**Figure 5:**
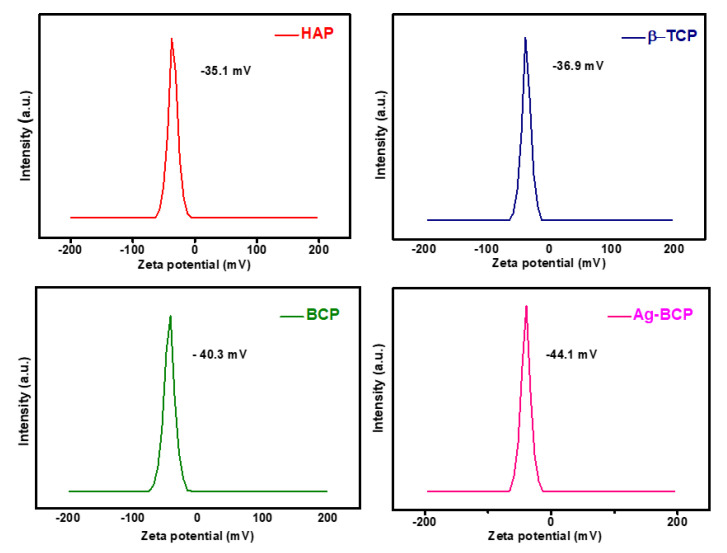
Zeta potential analysis of HAP, β-TCP, BCP, and Ag-BCP.

Figure 5 shows the zeta potential analysis of HAP, β-TCP, BCP, and Ag-BCP samples in an aqueous solution. These studies were used to estimate the colloidal stability and dispersions of bio-ceramics in solution. From the analysis, the zeta potential values of HAP, β-TCP, BCP, and Ag-BCP were obtained to be −35.1 mV, −36.9 mV, −40.3 mV, and −44.1 mV, respectively (Figure 5a–d). The zeta potential value obtained for pure HAP was greater than −30 mV compared to the literature report [31] and in the same way, the β-TCP sample with a value of −36.9 mV changed to −40.3 mV for BCP, indicating that the system arrived at a state of moderate stability. Further, for the Ag-BCP sample, the potential value of −44.1 mV was observed indicating the influential stability of the Ag NPs onto the surface of BCP.

**Figure 6:**
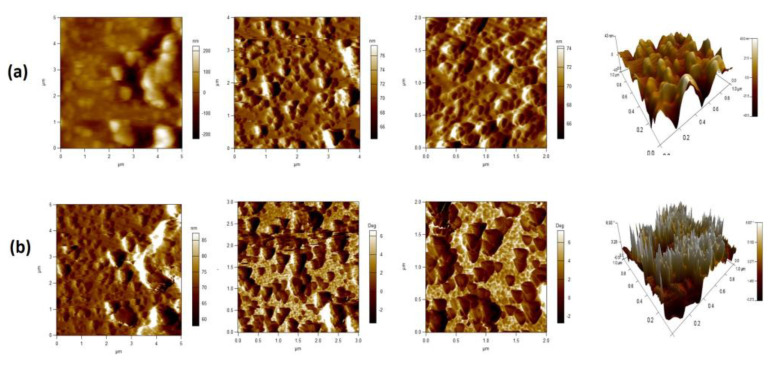
Surface topographical analysis for (**a**) BCP and (**b**) Ag-BCP.

The AFM provided topographic analyses of BCP and Ag-BCP samples and are shown in Figure 6. For the topographical examination, both samples in their powdered form were coated with Aluminum foil. As shown in Figure 6a, the topography of the BAP sample confirms the formation of homogeneously arranged agglomerated globules. Furthermore, the 2D image of Ag-BCP (Figure 6b) shows that the triangular shape is embedded on the globules representing Ag and globules for the BCP. Similarly, the 3D image of Ag-BCP shows the formation of highly crystalline homogenous peaks corresponding to Ag and are in good agreement with the 2D image peaks [32] (Table S1).

**Figure 7:**
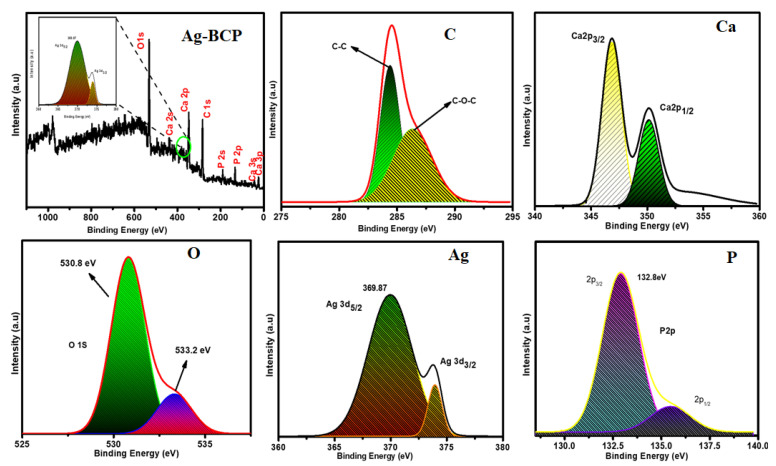
XPS spectroscopic analysis of Ag-BCP samples.

The XPS analysis was used to determine the elemental composition of Ag-BCP samples and the results are provided in Figure 7. The image provides the XPS spectrum along with the elemental peaks of C, Ca, O, Ag, and P. The spectrum of the elements O1s, P2p, Ca2p, Ag3d, and C1s are due to the adsorption of hydrocarbon impurities. The C-C component at 285 eV is used to calibrate the energy level. The spectrum of C1s shows two major peaks inclined at 284.95 eV for the non-oxygenated sp2 carbon ring (C-C) and at 286.5 eV for the sp3 (C-O) oxygenated functional group of carbon. Furthermore, the XPS of Ca2p shows two distinct peaks 347.35 and 351.23 eV corresponding to the Ca2p_3/2_ and Ca2p_1/2_, respectively. The XPS of O1s shows binding energies of 530.8 eV and 533.2 eV corresponding to -OH and P-O-P, respectively. Similarly, the prominent spectrum for Ag arises at 369.87 eV and 372.18 eV corresponding to 3d_5/2_ and 3d_3/2_. The observation of this Ag peak confirms the successful decoration of Ag NPs onto the surface of the BAP sample [33]. The phosphate spectra of P2p peaks at 132.8 eV and 135 eV correspond to P2p_3/2_ and P2p_1/2_ and confirm the success of HAP formation.

**Figure 8:**
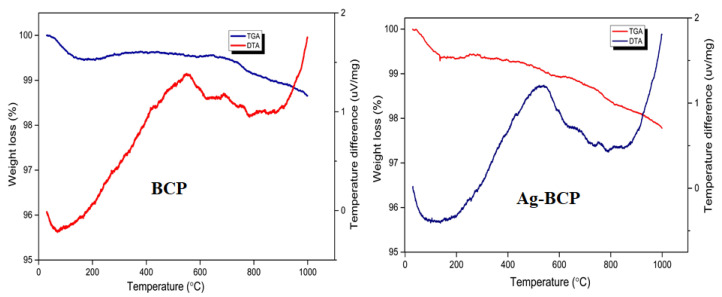
TGA and DTA analysis of BCP and Ag-BCP samples.

Figure 8 provides a comparison of the thermal stability of BCP and Ag-BCP samples as analyzed by TGA and DTA. The initial weight loss of up to ≈210 °C for both samples was due to the loss of moisture/adsorbed water (up to 200 °C) and lattice water (up to 650 °C). Above this, the occurrence of weight loss was seen in several stages between 200 °C and 900 °C, confirming the transformation of the HAP phase into a β-TCP phase in the BCP sample [34]. For the BCP and Ag-BCP samples, the total weight loss measured around 1000 °C was only 1% and 0.5%, respectively, thereby indicating that the Ag decoration reduced the thermal stability of BCP.

**Figure 9:**
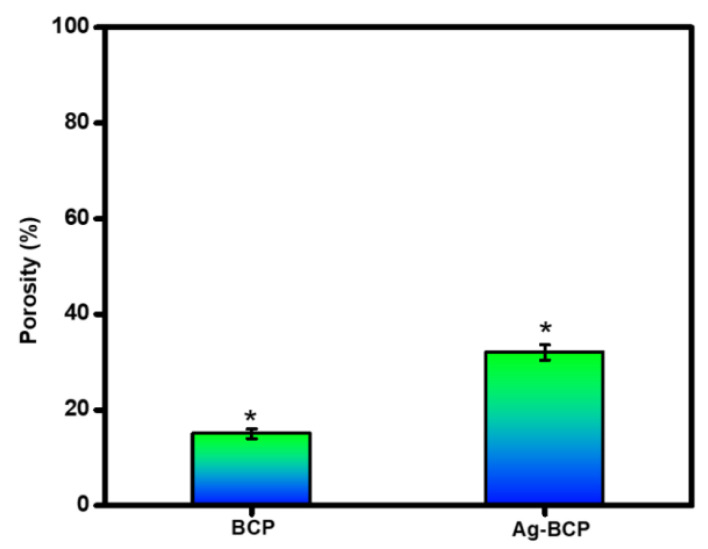
Porosity evaluation of BCP and Ag-BCP samples by the liquid displacement method. * denoted as statistical analyses were performed using a one-way analysis of variance (ANOVA) and the data presented are the mean ± standard deviation.

Figure 9 shows the comparison of porosity measurements of BCP and Ag-BCP samples. The porosity of bare BCP was observed around 15.20%. BCP samples decorated with Ag NPs showed an increase in porosity value at 32%. Such an observation of increased porosity values is due to the occurrence of chemical interactions of Ag NPs with the BCP matrices. This is the most useful factor in bone tissue engineering applications, as it can facilitate cell growth and migration, protein delivery to the cells, and preserve tissue volume with temporary mechanical function [35].

### 3.2. Studies of Bioactivity, Biodegradation, and Drug Release

**Figure 10:**
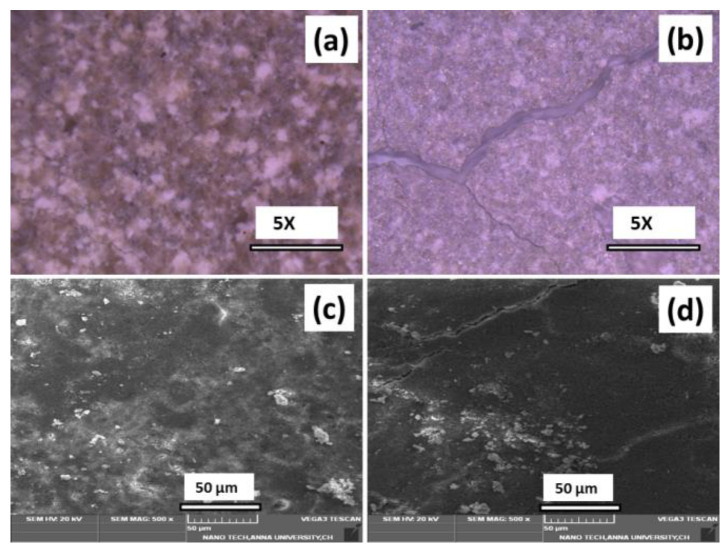
Bioactivity investigation recorded by the optical microscopic images for BCP (**a**) and Ag-BCP s (**b**) samples and the corresponding SEM images of BCP (**c**) and Ag-BCP (**d**).

Figure 10 shows the in vitro bioactivity studies of BCP and Ag-BCP samples as investigated by the amount of apatite formed on the sample’s surface. The sample’s morphological changes associated with the formation of surface masses were recorded by SEM. The materials were maintained in SBF for 14 days at 37 °C. The optical and SEM images of BCP (Figure 10a,c) show fibers with an anisotropic aspect of ~5–10 µm in length and 0.5–1.0 µm in width. However, the Ag-BCP sample (Figure 10b,d) witnessed the formation of an apatite layer at the surface of pellets and the newly formed layer comprised of tiny spherical particles of calcium phosphate crystals. This indicates that the Ag-BCP composite serves as a bioactive material with the ability to generate an apatite layer that can bond bones with implant materials [36].

**Figure 11:**
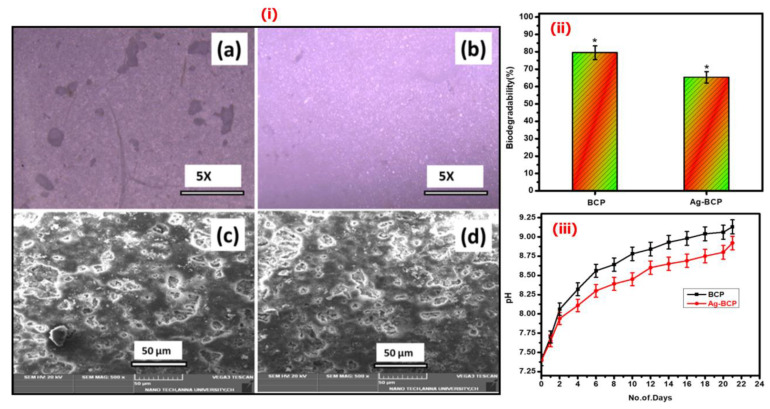
(**i**) Biodegradability investigation using optical microscopy (**a**,**b**) and SEM analysis (**c**,**d**) for the BCP and Ag-BCP samples. The degradability efficiency (%) evaluation is determined by the weight-loss method (* denoted as statistical analyses were performed using a one-way analysis of variance (ANOVA) and the data presented are the mean ± standard deviation) (**ii**) and a pH meter investigation (**iii**).

Optical microscopic and SEM images of BCP and Ag-BCP samples are employed to understand the biodegradation efficiencies linked to surface morphological changes followed by the incubation in SBF (14 days, 37 °C, see Figure 11(i)) For both samples, the surface roughness increased due to the degradation of material into the SBF medium. Furthermore, after 21 days in SBF, 79.45% of BCP and 65.25% of the Ag-BCP composite degraded (Figure 11(ii)). Further, the biodegradation behavior of BCP and Ag-BCP samples in tris-buffer is compared in Figure 11(iii). We observed an overall increase in the pH value after the sample’s immersion. This indicates that the BCP sample had an increased pH compared to Ag-BCP. Over 3 weeks, Ag-BCP had the slowest bio-dissolution, while for the BCP, the fastest mass decrease and high dissolution rates with pH increase were noted. This result indicates that both samples are highly biodegradable. Pure BCP (as against Ag-BCP) degrades fastest and the differences can be linked to the availability of solid Ag NPs in the tri-component system. This difference in biodegradation is well suited for bone tissue engineering applications as it helps to withstand mechanical stress and creates an encouraging environment for cell attachment and growth.

**Figure 12:**
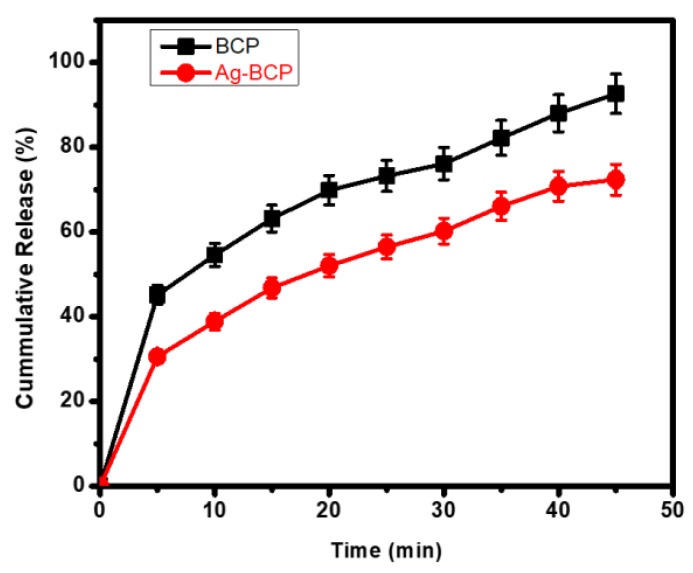
Comparison of CIP release behavior of BCP and Ag-BCP.

Figure 12 shows the pattern of CIP release from BCP and Ag-BCP samples under physiological conditions (PBS; pH 7.4). The analysis was carried out by measuring the optical absorption as a function of time. BCP exhibited an initial burst release of ~43% within the first 5 mins. In contrast, the Ag-BCP sample showed a relatively lower release of only 30%. Over 45 mins, BCP released ±92.65%, whereas Ag-BCP released only ±72.37% in a slow and controlled way. This difference is linked to the presence of Ag which supports the occurrence of heterogeneous oxidation reactions requiring the combined effects of dissolved oxygen and protons [36]. From the CIP release studies, we observed that the Ag-BCP has controlled release behavior due to its capacity to maintain the heterogenous particles of varying sizes, shapes, and phases.

### 3.3. In Vitro Antimicrobial and Cytocompatibility Studies

**Figure 13:**
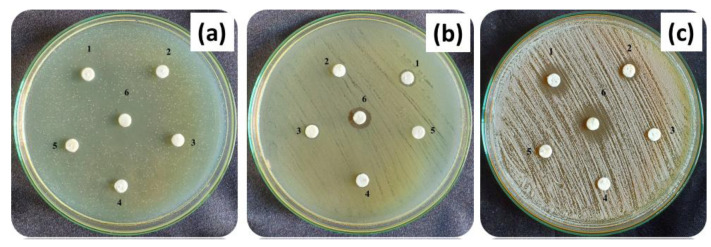
Antimicrobial investigation against (**a**) *S. aureus*, (**b**) *E. coli*, and (**c**) *C. albicans* (where 1. Ag-BCP, 2. HAP, 3. Β-TCP, 4. BCP, 5. DMSO, and 6. Standard).

HAP, β-TCP, BCP, and Ag-BCP samples were tested for their antibacterial activity against the Gram-positive *S. aureus*, Gram-negative *E. coli*, and the antifungal activity against *C. albicans* (shown in Figure 13a–c). The reason for selecting *S. aureus* for the studies is that it is responsible for the biofilm formation on bone implants. *E. coli* strains have a reducing capability towards BCP. From the comparison of results provided in Figure 13, the Ag-BCP sample performed almost equal to the standard (Std) in both antibacterial and antifungal activities. Ag-BCP had a moderate inhibitory effect against all bacteria or fungi during a 12 h culturing period. Among the two different bacterial cultures, Ag-BCP had the highest activity towards gram-positive bacteria (*S. aureus*) and fungi (*C. albicans*) where the ZoI was about 10 mm. Furthermore, gram-negative bacteria (*E. coli*) were inhibited in their growth with ZoI of 8 mm. The ZoI of all the samples (HAP, β-TCP, BCP, and Ag-BCP) against the tested microbial cultures are provided in Table 3. Ag-BCP shows better antimicrobial (antibacterial and antifungal) activity than that of the other three samples and thereby confirms the role played by the Ag NPs impregnated onto the BCP composite.

**Figure 14:**
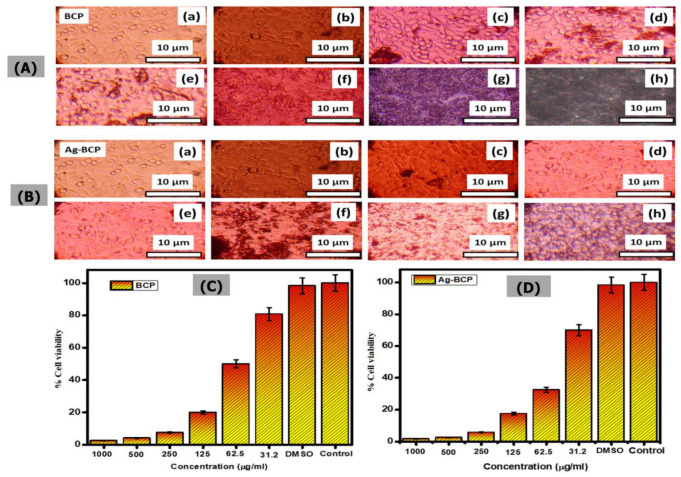
Optical microscopic images of L929 fibroblast cells under various concentrations of BCP (**A**) and Ag-BCP (**B**) ((**a**) for the control, (**b**–**h**) for the cell concentration in the range of 31.2 to 1000 µg/mL). The corresponding % cell viability changes for BCP (**C**) and Ag-BCP (**D**) in L929 cells.

Figure 14 provides a comparison of in vitro cytocompatibility studies of BCP and Ag-BCP samples when tested on mouse fibroblast L929 cells at various concentrations. We performed an MTT assay carried out with BCP and Ag-BCP samples. Both cells exhibited a significant reduction in the number of cells (detected via their absorption recorded at 450 nm) with an increase in treatment dosage from 31.2 to 1000 µg/mL. When changing the concentration from 31.2 to 62.5 µg/mL, the cell viability % decreased from 80.8 to 50% for the BCP sample and 70 to 32.5% for the Ag-BCP. This determines the IC_50_ value of BCP and Ag-BCP to be 62.5 and 46.8 µg/mL, respectively.

## 4. Conclusions

In conclusion, the present study deals with the synthesis, characterization, and testing of BCP and Ag-BCP composites for antimicrobial, drug delivery, and biodegradable characteristics. Bioceramic composites (Venice Mestre, Italy) were characterized for improved interconnectivity, porosity, moderate compressive strength, and biocompatibility where all of these properties are beneficial for bone tissue engineering applications. The Ag-BCP composite was formed by the decoration of Ag NPs with the bioceramic BCP base. The composite maintained its BCP structural framework and at the same time, the porous network structure was formed without compromising its basic characteristics. Nonetheless, the compressive strength and thermal stability increased after Ag loading onto the BCP. Furthermore, the composites showed a difference in pH values in SBF solution, and for the Ag-BCP, the biodegradation rate was reduced. Based on the cumulative results, the Ag-BCP composite would serve as a potential candidate for the efficient growth of damaged or defective bone parts in tissue engineering applications.

## Figures and Tables

**Table 1:** FTIR spectral data for (a) HAP, (b) β-TCP, (c) BCP, and (d) Ag-BCP samples.

Vibrational Frequency (cm^−1^)	Band Assignment			
	HAP	β-TCP	BCP	Ag-BCP
3572, 630	-OH group	-OH group	-OH group	-OH group
2920	-	-	-	Glucose-assisted Ag NPs (C-H) stretching
1032, 1098, 1133	Asymmetric stretching vibrations of the P–O bonds	1133 is absent	Asymmetric stretching of the P–O bonds	Asymmetric stretching of the P–O bonds
1037	-	PO_4_^3−^ ions found in β-TCP	PO_4_^3−^ ions found in β-TCP	PO_4_^3−^ ions found in β-TCP
926	Symmetric stretching (υ_1_) of P–O bond from PO_4_^3−^ group	-	Symmetric stretching (υ_1_) of P–O bond of PO_4_^3−^ group	Symmetric stretching (υ_1_) of P–O bond of PO_4_^3−^ group
960	-	Symmetric stretching (υ_1_) of P–O bond of PO_4_^3−^ group	Symmetric stretching (υ_1_) of P–O bond of PO_4_^3−^ group	Symmetric stretching (υ_1_) of P–O bond of PO_4_^3−^ group
730	Owing to H_2_O	-	-	Owing to H_2_O
631	Liberational OH group	Liberational OH group	Liberational OH group	Liberational OH group
602, 560	Phosphate bands (υ_4_)	Vibrational bands of PO_4_^3−^	Phosphate bands (υ_4_)	Phosphate bands (υ_4_)
498, 452	Phosphate bands (υ_2_)	Phosphate bands (υ_2_)	Phosphate bands (υ_2_)	Phosphate bands (υ_2_)

**Table 2:** Raman spectral data of (a) HAP, (b) β-TCP, (c) BCP, and (d) Ag-BCP.

Frequency of Vibration (cm^−1^)	Band Assignments			
	HAP	β-TCP	BCP	Ag-BCP
1364	-	-	-	Gluconic acid
1056 1090	Asymmetric stretching vibrations (υ_3_) of PO_4_^−3^	Asymmetric stretching vibrations (υ_3_) of PO_4_^−3^	Asymmetric stretching vibrations (υ_3_) of PO_4_^−3^	Asymmetric stretching vibrations (υ_3_) of PO_4_^−3^
966	Symmetric stretching vibrations (υ_1_) of PO_4_^−3^ group	-	Symmetric stretching vibrations (υ_1_) of PO_4_^−3^ group	Symmetric stretching vibrations (υ_1_) of PO_4_^−3^ group
964, 948	-	Internal vibrations of β-TCP in BCP	Internal vibrations of β-TCP in BCP	Internal vibrations of β-TCP in BCP
432, 445	Symmetrical bending (υ_2_)	Symmetrical bending (υ_2_)	Symmetrical bending (υ_2_)	Symmetrical bending (υ_2_)
572, 598	Asymmetric bending (υ_4_) vibrations of PO_4_^−3^	Asymmetric bending (υ_4_) vibrations of PO_4_^−3^	Asymmetric bending (υ_4_) vibrations of PO_4_^−3^	Asymmetric bending (υ_4_) vibrations of PO_4_^−3^

**Table 3:** Zone of inhibition (mm) data of bioceramic samples treated against *E. coli*, *S. aureus*, and *C. albicans*.

Microorganism	Zone of Inhibition (mm)					
	Ag-BCP	HAP	β-TCP	BCP	DMSO	Std (20 µL)
*E. coli*	8 ± 1.15	-	-	-	-	11 ± 1.75
*S. aureus*	10 ± 2.2	-	-	-	-	17 ± 1.2
*C. albicans*	10 ± 1.5	-	-	-	-	12 ± 1.0

## Data Availability

Not applicable.

## Supplementary Materials

The following supporting information can be downloaded at: https://www.mdpi.com/article/10.3390/antibiotics11121780/s1, Table S1: polydispersity index.
