# Supplementary material for: Biosynthesised Silver Nanoparticles Loading onto Biphasic Calcium Phosphate for Antibacterial and Bone Tissue Engineering Applications
Source: Antibiotics (Basel). 2022 Dec 8;11(12):1780. doi: 10.3390/antibiotics11121780 (PMC9774414; doi:10.3390/antibiotics11121780)
Supplement: Supplementary file 1 [file antibiotics-11-01780-s001.zip › antibiotics-2049187-supplementary.pdf]

Table S1 polydispersity index

S. No	Sample	Mean (nm)	S.D(nm)	PdI	Dispersive medium
1.	HAP	391.6nm	77.3nm	0.197	Monodispersive
2.	β-TCP	276.4nm	22.8nm	0.082	Monodispersive
3.	BCP	222.5nm	21.3nm	0.095	Monodispersive
4.	Ag-BCP	93.9	7.4nm	0.078	Monodispersive
